# The Effect of Attractive Interactions and Macromolecular Crowding on Crystallins Association

**DOI:** 10.1371/journal.pone.0151159

**Published:** 2016-03-08

**Authors:** Jiachen Wei, Jure Dobnikar, Tine Curk, Fan Song

**Affiliations:** 1 State Key Laboratory of Nonlinear Mechanics (LNM), Institute of Mechanics, Chinese Academy of Sciences, 100190, Beijing, China; 2 International Research Center for Soft Matter, Beijing University of Chemical Technology (BUCT), 100029, Beijing, China; 3 The University Chemical Laboratory, University of Cambridge, Lensfield Road, CB2 1EW, Cambridge, United Kingdom; Tsinghua University, CHINA

## Abstract

In living systems proteins are typically found in crowded environments where their effective interactions strongly depend on the surrounding medium. Yet, their association and dissociation needs to be robustly controlled in order to enable biological function. Uncontrolled protein aggregation often causes disease. For instance, cataract is caused by the clustering of lens proteins, i.e., crystallins, resulting in enhanced light scattering and impaired vision or blindness. To investigate the molecular origins of cataract formation and to design efficient treatments, a better understanding of crystallin association in macromolecular crowded environment is needed. Here we present a theoretical study of simple coarse grained colloidal models to characterize the general features of how the association equilibrium of proteins depends on the magnitude of intermolecular attraction. By comparing the analytic results to the available experimental data on the osmotic pressure in crystallin solutions, we identify the effective parameters regimes applicable to crystallins. Moreover, the combination of two models allows us to predict that the number of binding sites on crystallin is small, i.e. one to three per protein, which is different from previous estimates. We further observe that the crowding factor is sensitive to the size asymmetry between the reactants and crowding agents, the shape of the protein clusters, and to small variations of intermolecular attraction. Our work may provide general guidelines on how to steer the protein interactions in order to control their association.

## Introduction

The fiber cells of eye lens contain no organelles, but almost exclusively a dense suspension of proteins, namely, the *α*, *β* and *γ*-crystallins [1, 2]. In the nucleus of human eye lens, the predominant proteins are *γ*-crystallins [3, 4] (for brevity, we will use “crystallins” instead of “*γ*-crystallins” in the rest of the manuscript). The moderate attractions between crystallins allow their self-assembly into organized patterns [5, 6] with short-range spatial order, which plays an important role in maintaining the transparency of the lens. Recent studies [7–11] have shown that prenatal or age-related genetic mutations associated to cataract can lead to modified intermolecular interactions, resulting in crystallin association and cross-linked structures with a lower degree of local order producing the blur in the lens [12, 13]. Modified crystallins change the osmotic balance of the lens inner cells by agglomerating into larger clusters, thereby reducing the excluded volume that directly leads to the decrease of the osmotic pressure [14–16]. In order to compensate for such pressure loss, the tissue must expel water or increase the concentration of the non-aggregated proteins. Furthermore, if the aggregation becomes so extensive that the osmotic pressure cannot be restored, the biochemical equilibrium within the eye lens is destroyed and the cells deform and rupture, leading to cataract. Due to its prevalence and serious impact on the quality of life, cataract has been extensively studied, however its molecular origins [1] are to date not fully understood—hindering efficient disease prevention or treatment.

One of the major open questions is the relation between the microscopic protein-protein interactions and the thermodynamic properties of crowded crystallin solutions. Das *et*. *al*. [17, 18] calculated the stability of crystallins by all-atom molecular simulations, showing that crystallin attraction can result in protein polymerization. Such detailed simulations are prohibitively expensive when addressing the effect of molecular crowding. Coarse grained models of hard spheres with short-range square-well attraction have been applied to study phase separation [19–21] and physical aggregation [6, 22] of crystallins, revealing that moderate intermolecular attraction is crucial in maintaining the thermodynamic stability of the system. Here we focus on how the association-dissociation equilibrium of a pair of proteins embedded in a crowded environment depends on the magnitude of their attraction. We adopt the scaled particle theory (SPT) [23] approach that has been previously used to study polymerization diseases of proteins, such as sickle cell and Alzheimer’s disease [24, 25]. The protein attraction is modelled in two ways: by thermodynamic perturbation model (TPM) [26] and by chemical binding model (CBM) [27], both of which separate intermolecular interactions into contributions from steric depletion and chemical attraction. Due to the simplicity of the models, we can address the problem analytically and discuss the generic features of association equilibria in crowded attractive systems.

## Methods

**Fig 1:**
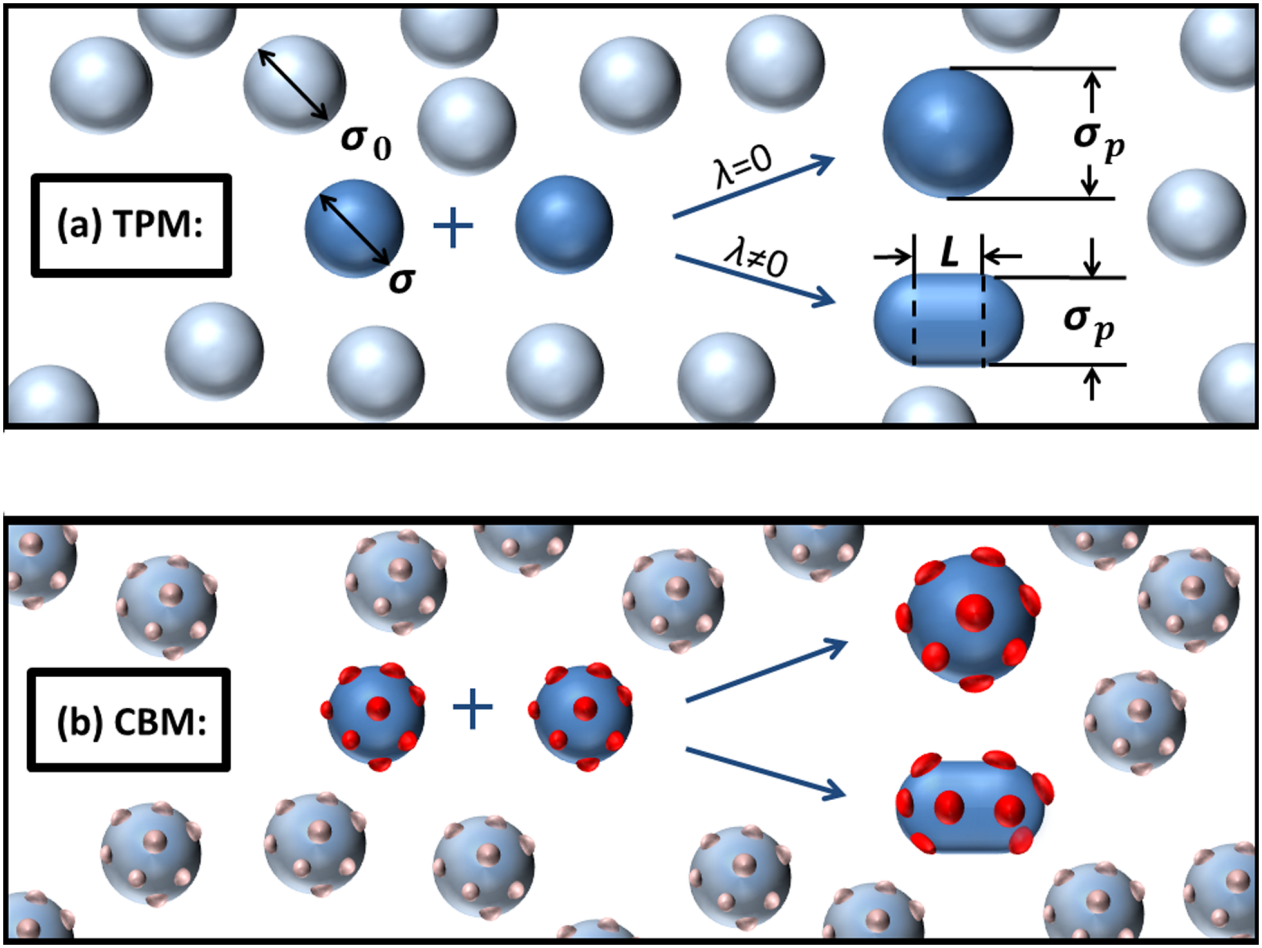
Association of two crystallins in macromolecular crowding. Sketch of two crystallins as reactants polymerising into a dimer as product for (a) thermodynamic perturbation model (TPM), where blue spheres represent crystallins with isotropic intermolecular attraction, and (b) chemical binding model (CBM), where blue spheres and red points respectively represent crystallins with steric repulsion and binding sites with chemical attraction. The general shape of the product is a spherocylinder with asphericity λ.

The SPT characterizes thermodynamic properties of macromolecular or colloidal solutions by describing them as effective hard-core convex particles [28]. We consider a crowded environment with spherical particles (crowders) of diameter *σ*_0_ within which there are two spherical crystallins as reactants of diameter *σ* = 3.6 nm. We denote the ratio of reactant and crowder sizes as *ς* = *σ*/*σ*_0_. We will focus on the ratio *ς* = 1, i.e. the reactants and the crowders are identical crystallins. The effect of size polydispersity is addressed in the *Online SI*. The reactants associate into a product (Fig 1) with volume *πσ*^3^/3 whose shape depends on the microscopic details of the protein-protein interactions. We model the products as spherocylinders with the length *L* and the diameter of hemispherical caps *σ*_*p*_ (see Fig 1). The deviation from the spherical shape can be defined by an asphericity parameter *λ* ≡ *L*/*σ*_*p*_. Taking the volume conservation into account, we have

$$\begin{array}{c} \sigma_{p}=\sigma \sqrt[{3}]{\frac{4}{3\lambda +2}}\;,\;L=\lambda \sigma_{p}\;. \end{array}$$ (1)

For spherical product (λ = 0) we have *L* = 0 and $\sigma_{p}=\sqrt[{3}]{2}\sigma$.

### Crowding factor

The crowding factor (or non-ideality factor), Γ, measures the contribution of crowders to the association equilibria of the two reactants:

$$\begin{array}{c} \Gamma =\frac{K}{K_{0}}, \end{array}$$ (2)

where *K*_0_ and *K* denote the equilibrium association constants in dilute and crowded environment, respectively, and are related to the activity coefficients *γ*_r_ and *γ*_p_ for reactant and product:

$$\begin{array}{c} \ln K-\ln K_{0}=2\ln\gamma_{r}-\ln\gamma_{p}\;. \end{array}$$ (3)

Combining Eqs 2 and 3, we obtain:

$$\begin{array}{c} \Gamma =\frac{\gamma_{r}^{2}}{\gamma_{p}}. \end{array}$$ (4)

The activity coefficient, *γ* (for either reactant or product), is associated to the work of inserting another reactant/product particle into the sea of crowders. For system of hard-spheres, according to the SPT, we have:

$$\begin{array}{c} \ln\gamma^{st}=-\ln\left(1-\phi \right)+A_{1}\frac{\phi }{1-\phi }+A_{2}{\left(\frac{\phi }{1-\phi }\right)}^{2}+A_{3}{\left(\frac{\phi }{1-\phi }\right)}^{3}, \end{array}$$ (5)

where *ϕ* is the packing fraction of the system, and [27, 29]

$$\begin{array}{c} \begin{array}{c} A_{1}=\varsigma^{3}+3\varsigma^{2}+3\varsigma +1.5\lambda \left(\varsigma^{2}+2\varsigma +1\right)\\ A_{2}=3\varsigma^{3}+4.5\varsigma^{2}+4.5\lambda \left(\varsigma^{2}+\varsigma \right)\\ A_{3}=3\varsigma^{3}+4.5\lambda \varsigma^{2}\;. \end{array} \end{array}$$ (6)

For the reactants, *ς* = 1 and λ = 0, thus *A*_1_ = 7, *A*_2_ = 7.5 and *A*_3_ = 3. For the products, *ς* = *σ*_p_/*σ*_0_.

The total activity in the case of associating proteins is a sum of the steric part, *γ*^*st*^, due to the hard-sphere part of the interactions and the “chemical” part, i.e. the contribution of the attractive interactions, *γ*^*ch*^:

$$\begin{array}{c} \ln\gamma =\ln\gamma^{st}+\ln\gamma^{ch}\;. \end{array}$$ (7)

Below we describe the two models applied in order to estimate *γ*^*ch*^: TPM and CBM. The TPM describes the protein-crowder attraction by thermodynamic perturbation theory with orientational average approximation (Fig 1(a)). The CBM treats it as binding between crystallins with nonspecific binding sites (Fig 1(b)). Both approaches lead to predictions of association constants compatible with existing experimental data and numerical simulation for various types of globular proteins [26, 27, 30–33]. We therefore use the SPT in combination with TPM or CBM to determine the activity coefficient, crowding factor and osmotic pressure of dense attractive crystallin suspensions. The osmotic pressure Π is evaluated as [34]:

$$\begin{array}{c} \Pi =RT\left(\rho +\int_{0}^{\rho^{*}}\rho \frac{d\ln\gamma }{d\rho }d\rho \right), \end{array}$$ (8)

where *R* is the molar gas constant, *T* the absolute temperature and *ρ* = 6*ϕ*/*πσ*^3^ the number density.

### Thermodynamic perturbation model (TPM)

In the TPM [26], *γ*^*ch*^ is approximated using thermodynamic perturbation theory and orientational average as

$$\begin{array}{c} \ln\gamma^{ch}=-\rho \epsilon \text{S}\left[\delta \text{r}+\left(g_{0}^{max}-1\right)\theta \right] \end{array}$$ (9)

where *ϵ* (in unit of *k*_*B*_*T*) is the orientationally averaged depth of the attraction minimum, and *S* is the surface area of the protein. *δr* is the range of the attraction. The value *δr*/*σ* = 0.2 was found to be the suitable ratio as compared to Monte Carlo simulation results for a range of globular proteins [26], therefore we adopt this ratio here as well. Finally, $g_{0}^{max}$ is the peak value of the radial distribution function, and *θ* = (2^1/6^ − 1)*σ*/2 is its decay range. Here we choose the Carnanhan-Starling [35] equation of state to derive the relation between $g_{0}^{max}$ and the volume fraction *ϕ*:

$$\begin{array}{c} g_{0}^{max}=\frac{1-\phi /2}{{\left(1-\phi \right)}^{3}}\;. \end{array}$$ (10)

### Chemical binding model (CBM)

Within the CBM [27], *γ*^*ch*^ is calculated by treating intermolecular attraction as chemical binding between crystallins (Fig 1(c)). Assume that the binding is nonspecific, we have [27]

$$\begin{array}{c} \ln\gamma^{ch}=-n_{s}\ln\left(1+K\frac{\gamma_{r}^{st}\gamma_{b}^{st}}{\gamma_{r,b}^{st}}\phi \right), \end{array}$$ (11)

where $\gamma_{b}^{st}$ and $\gamma_{r,b}^{st}$ denote the steric repulsive part of activity coefficient for crowder and reactant-crowder complex, respectively. In our case, since both reactants and crowders are crystallins, we have $\gamma_{b}^{st}=\gamma_{r}^{st}$ and $\gamma_{r,b}^{st}=\gamma_{p}^{st}$. *n*_*s*_ = *αS* is the number of binding sites, where *α* represents a temperature-independent coefficient that reflects the density of the binding sites. The binding constant, *K*, which is temperature-dependent, reflects the strength of the attraction between two binding sites.

The *γ* − *ϕ* and Γ − *ϕ* relations at different value of *ϵ* for TPM or *K* for CBM are respectively presented in S1 and S2 Figs. Also we note that, besides these two models, there is still another effective hard-sphere model introduced by Minton [36]. See S3–S6 Figs for more discussions.

## Results and Discussion

### Comparison with experimental data

**Fig 2:**
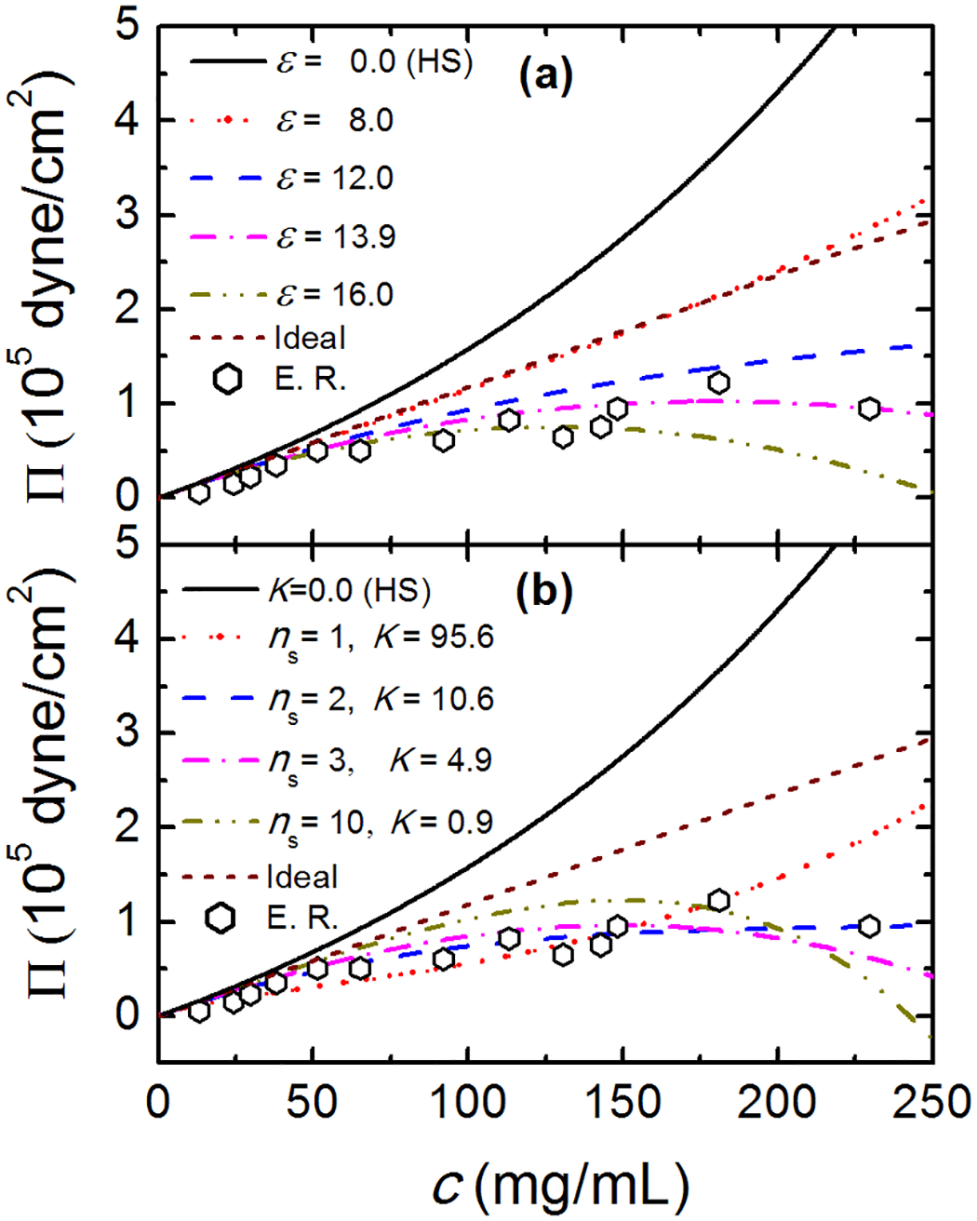
The osmotic pressure, Π, as a function of protein concentration, c. (a) the Π − *c* relation for TPM at different *ϵ*. (b) best fitting Π − *c* curves for CBM with different combinations of *n*_*s*_ and *K*. Black symbols denote experimental results derived from the work of Tardieu *et*. *al*. [15, 37]. In order to compare analytic with experimental results, we set *T* = 298.15*K*.

We first focus on the case where the product has a spherical shape (λ = 0). The osmotic pressure for TPM at different average minimum attraction, *ϵ*, is shown in Fig 2(a). It is observed that the value of Π sensitively depends on *ϵ*, especially at higher protein concentration *c*. For *ϵ* < 12, the value of Π increases monotonically with the increase of *c*. Note that when *ϵ* = 0, we obtain the osmotic pressure for hard-spheres based on SPT. For *ϵ* > 13, with the increase of *c*, the value of Π first increases and then decreases, due to the existence of strong intermolecular attraction. The case *ϵ* = 8.0 best describes the osmotic pressure of an ideal solute Π_*i*_ = *ρRT*.

**Fig 3:**
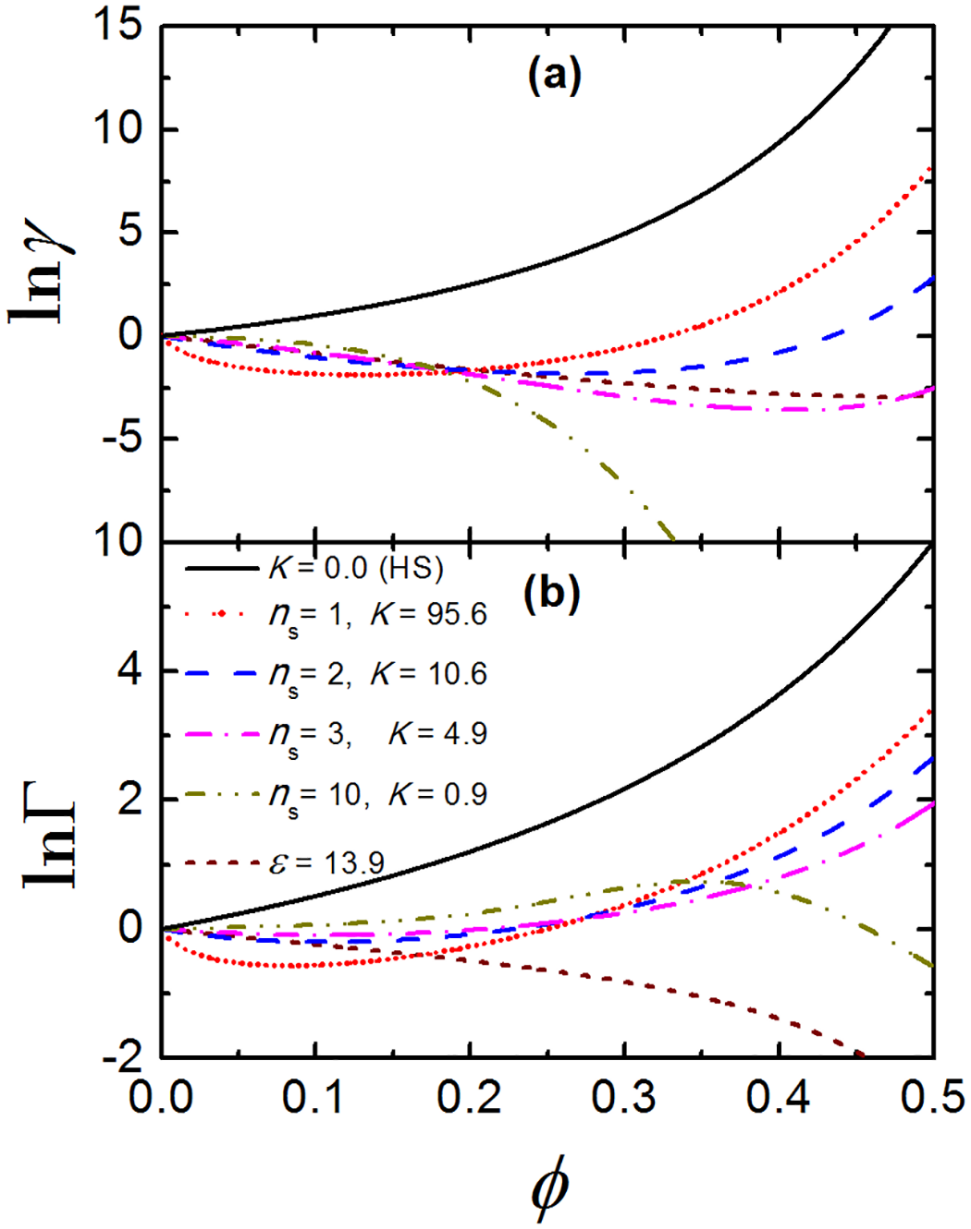
The activity coefficient and crowding factor for crystallins. (a) The activity coefficient, *γ*, and (b) the crowding factor, Γ, as a function of packing fraction, *ϕ*, for TPM with *ϵ* = 13.9 and CBM with different combinations of *n*_*s*_ and *K* that best fit the experimental data for osmotic pressure of crystallins.

From Fig 2(a) we also note that the experimental values [15, 37] of Π are always smaller than Π_*i*_, due to the attractive interactions between crystallins. The optimal parameter value *ϵ* = 13.9 (where almost quantitative agreement is observed) is obtained by least-square fitting procedure. In order to determine the best fitting parameters (*n*_*s*_ and *K*) for CBM, we first fix the value of *n*_*s*_, and determine the corresponding value of *K* by least square fitting. Several combinations (*n*_*s*_, *K*) of number and strength of the binding sites can qualitatively describe the experimental data, however, the quantitative agreement for the entire density range is obtained only for the number of binding sites between 2 (*n*_*s*_ = 2 and *K* = 10.6) and 3 (*n*_*s*_ = 3 and *K* = 4.9). A further consistency test is comparing the activity coefficient *γ* of TPM and CBM. Since the TPM has been thoroughly compared with Monte Carlo simulations for globular proteins other than crystallins [26], we expect that the activity coefficient predicted by TPM is close to the actual values for crystallins. In Fig 3(a) we compare the results for the optimal TPM case with *ϵ* = 13.9 and different combinations of CBM parameters. Again, the number of binding sites between 2 and 3 is a best match between the models, while the examples for a single strong bond and for 10 very weak bonds are qualitatively different. This gives us further confidence to conclude that the number of binding sites for crystallin—crystallin interaction is 2 or 3. This prediction is different from what was earlier anticipated [6, 22] and is an important insight into the crystallin structure derived from simple theoretical modeling combined with molecular dynamics simulations and experimental measurements.

### Crowding factor

Next, we investigate how crowding factor, Γ, depends on the volume fraction *ϕ* for TPM and CBM, as illustrated in Fig 3(b). For TPM with *ϵ* = 13.9, ln Γ is negative and it decreases with increasing *ϕ*, which indicates that the macromolecular crowding favors the dissociation of the crystallins. Quite on the contrary, we find the sign of ln Γ for CBM depends on *ϕ*. For instance, ln Γ is negative at small *ϕ* while positive at higher *ϕ* for CBM with *n*_*s*_ = 1. When *n*_*s*_ = 2 or 3, which best fits the experimental data for osmotic pressure of crystallins, we observe ln Γ ∼ 0.0 for any given *ϕ* less than 0.3. This means that for CBM the crystallins are more or less in equilibria at dilute and moderate concentrations, which confirms that intermolecular attraction can help maintain the association equilibria of crystallins. In the case of many weak bonds (*n*_*s*_ = 10), however, such relation is reversed: ln Γ first increases and reaches its peak value around *ϕ* = 0.35; when *ϕ* is further increased, ln Γ decreases and becomes negative.

Interestingly, although the activity coefficients for the best fitting parameters of both models (*ϵ* = 13.9 for TPM, and *n*_*s*_ = 2 and *K* = 10.6 for CBM) are quite similar, the corresponding crowding factors are qualitatively very different. The activity coefficients only differ in the chemical part ln *γ*^*ch*^ (see Eqs 9 and 11), thus the different behavior can only originate from differences in ln *γ*^*ch*^ of the products. Qualitatively similar behavior of the crowding factor seems to be obtained only in the limit of large number of weak bonds, which, however, is not a good description for crystallin suspension. The two models therefore, despite describing well the osmotic pressure data, predict qualitatively different crowding effects for crystallins. It is reasonable to expect that CBM is more accurate than TPM for proteins with highly orientational attraction, since the former reflects the competition between the decrease in surface area and the increase in the attractive strength. However, new experiments or atomistic simulations should be performed in order to confirm this claim. It must also be noted that many-body effects are not regarded in any of the model, therefore they are generally applicable at dilute and moderate protein concentrations, i.e., *ϕ* ≲ 0.4 [28]. In what follows, we will use the optimal parameter values for both models, i.e. *ϵ* = 13.9 for TPM, and *n*_*s*_ = 2, *K* = 10.6 for CBM and compare their predictions in more detail.

The ln Γ − *ϕ* relation in weak intermolecular attraction, and the effect of number of binding sites on crowding factor for CBM are respectively presented in S7 and S8 Figs. We also note that the magnitude of the binding constant *K* and the average minimum attraction *ϵ* in TPM are related quantities. In S9 Fig the relation between both constants, i.e., the values of *K* and *ϵ* such that the activity coefficient calculated from both models is the same, is depicted showing an expected linear dependence at low densities and deviations from it in more crowded environment.

### The effect of product geometry

**Fig 4:**
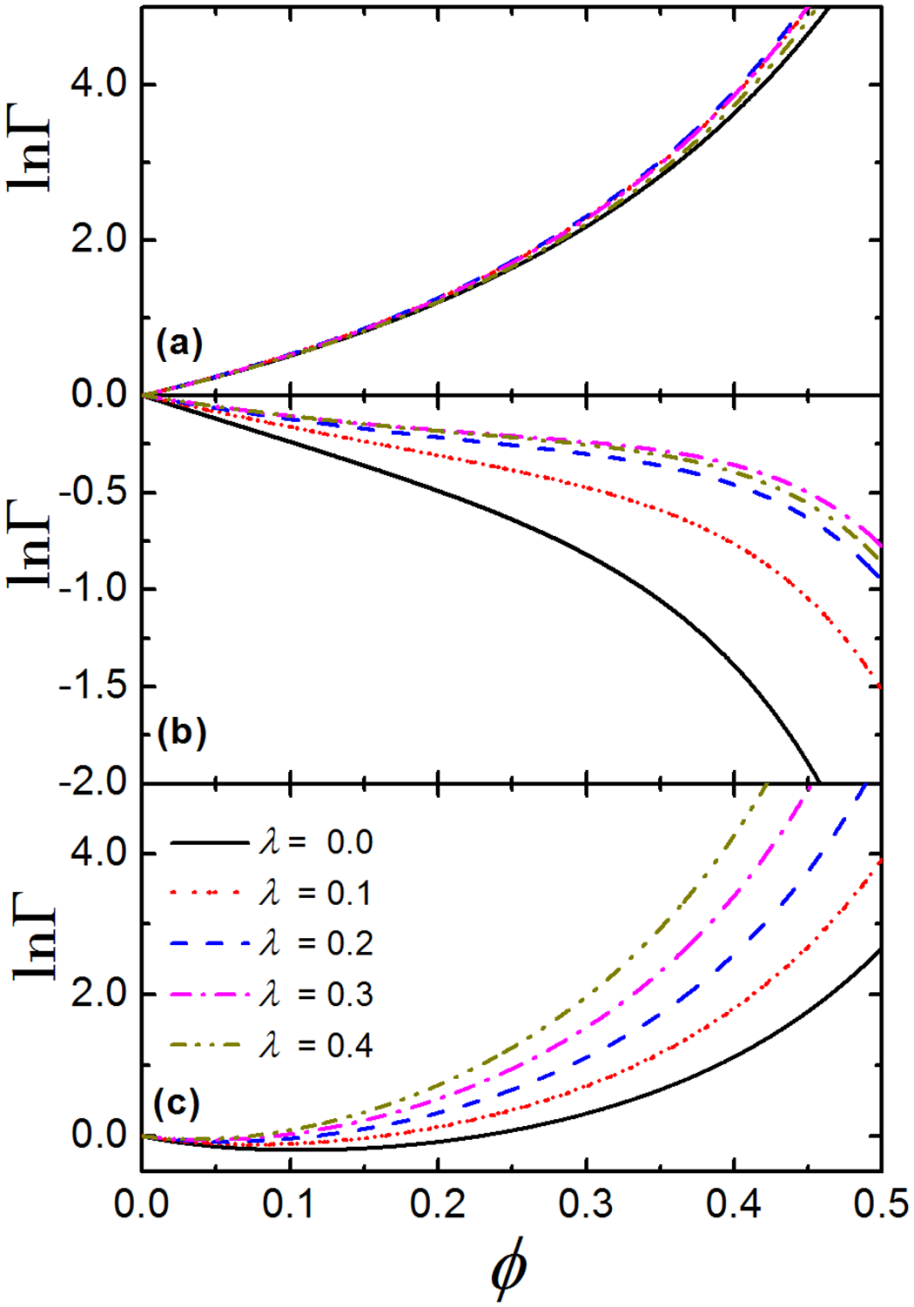
The influence of product shape on crowding factor of crystallins. The crowding factor, Γ, as a function of packing fraction, *ϕ*, at different value of asphericity parameter, λ, for (a) hard spheres, (b) TPM with *ϵ* = 13.9 and (c) CBM with *n*_*s*_ = 2 and *K* = 10.6.

Fig 4 presents ln Γ as a function of *ϕ* at different value of asphericity parameter, λ. According to the results of NMR spectroscopy [1], the best fitting value of asphericity parameter for crystallin dimers is approximately λ = 0.3. We observe that, for system without attraction, when λ increases from 0.0 to 0.4 (which is tantamount to increase the length of the cylinder part of product, *L*, from 0.0 to about 0.43), the ln Γ − *ϕ* relation is barely changed, see Fig 4(a). However, for TPM with *ϵ* = 13.9 (Fig 4(b)), ln Γ increases with the increase of λ at fixed *ϕ*, which indicates that crystallins are more likely to aggregate as the product becomes more nonspherical. This is because the surface area of the product would increase with the increase of λ, which directly decreases the activity coefficient of the product. Moreover, we notice that, even at very high packing fraction, ln Γ can hardly increase with the increase of λ when λ > 0.2, due to the cancelation effect of the increase of ln *γ*^*st*^ and the decrease of ln *γ*^*ch*^ for product. Fig 4(c) shows the ln Γ − *ϕ* relation at different λ for CBM with *n*_*s*_ = 2 and *K* = 10.6. We can see that, in dilute environment (*ϕ* < 0.2) ln Γ < 0 and it is slightly larger for larger λ at the same *ϕ*. However, at higher density, ln Γ is positive and it becomes more sensitive to the value of λ, since ln *γ*^*ch*^ for product is significantly decreased with the increase of asphericity of the product. For both TPM and CBM, one thing in common is that at dilute and moderate density, the value of ln Γ only slightly increased by changing λ from 0.0 to 0.4 at same *ϕ*, which means that the shape of the product has a limited impact on the association equilibria of crystallins in crowded environment.

We note that the crowding factor and the activity coefficient depend also on the reactant-crowder size ratio, *ς*. This effect, which is important in order to understand the association in polydisperse systems, is explored in more detail in S10 and S11 Figs.

### Entropy-enthalpy compensation

**Fig 5:**
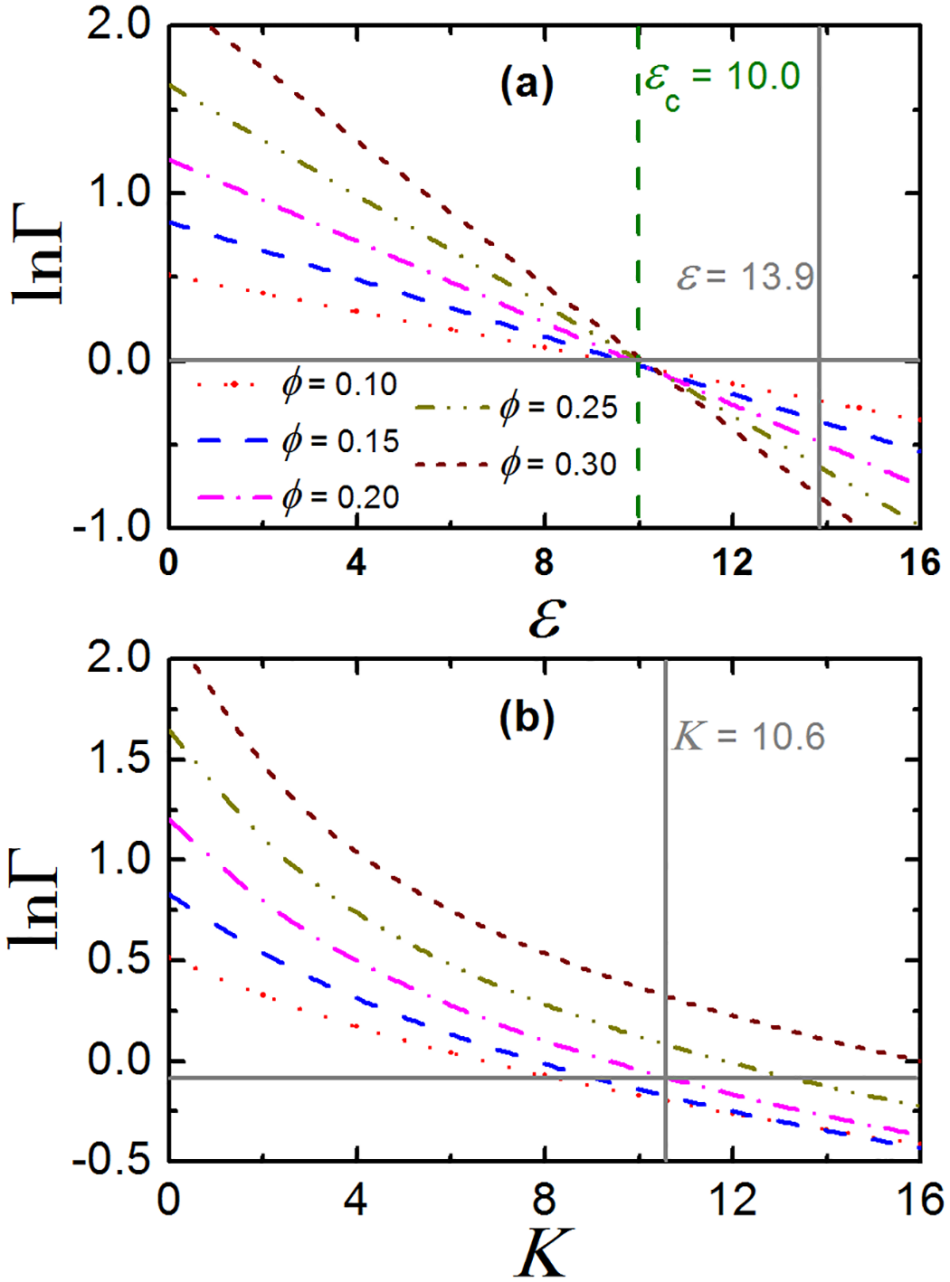
Crossover behavior in crowding factor. The crowding factor, Γ, as a function of average minimum attraction, *ϵ*, at different packing fraction, *ϕ*, for TPM. (b) Γ as a function of *ϵ* at different *ϕ*, for CBM. The horizontal solid line denotes ln Γ = 0.0. The vertical solid and dashed line respectively represents the fitting values of *ϵ* as well as *K* for crystallins, and the critical attraction at which ln Γ = 0.0 is achieved.

When ln Γ = 0, steric repulsion and chemical attraction between proteins are canceled out and effectively the crowded environment has no impact on the association equilibria of the proteins. This effect is called entropy-enthalpy compensation [26]. To determine the critical value of the fitting parameters (*ϵ*_*c*_ for TPM and *K*_*c*_ for CBM) at which entropy-enthalpy compensation is achieved (ln Γ = 0), here we present the crossover behavior of crowding factor in Fig 5. For TPM, we observe ln Γ decreases linearly with the increase of *ϵ* at fixed packing fraction, see Fig 5(a), which is in accord with former studies on association equilibria of other types of proteins [30, 31]. The critical average minimum attraction *ϵ*_*c*_ ≈ 10.0, which is almost independent on the value of *ϕ*. In addition, we find that at *ϵ* = 13.9, ln Γ is negative for any given *ϕ*, indicating that macromolecular crowding help stabilize the monodispersity of the crystallins.

The crossover behavior of crowding factor for CBM is quite different from that for TPM (note that here we still fix number of binding sites *n*_*s*_ = 2), see Fig 5(b). First, we find the ln Γ − *K* relation is no more linear at fixed packing fraction *ϕ*. Moreover, we find the critical binding constant *K*_*c*_, at which ln Γ = 0, is of different value for different *ϕ*. This can also be inferred from the ln Γ − *ϕ* relation in S2 Fig, since ln Γ fluctuates a lot at moderate and high packing fraction. For *K* = 10.6 (the fitting value for crystallins), the sign of ln Γ depends on the *ϕ*, which suggests the association equilibrium is sensitive to the concentration of crystallins. Nevertheless, we find ln Γ ∼ 0.0 when *ϕ* = 0.2, at which entropy-enthalpy compensation is achieved.

## Conclusion

By comparing the osmotic pressure calculated theoretically with two models and measured experimentally, we have identified the parameter regime in the SPT describing the attraction strength as well as number of binding sites for crystallins in the eye lens. The approach is general and valid for other globular proteins, however, different parameter regimes might be relevant for different molecules. Here we predict that the association equilibria of crystallins in eye lens are very sensitive to the protein concentration and the intensity of intermolecular interaction: slight modification of the interaction, or protein concentration in crowded environment, can result in extensive association or disassociation of crystallins, which may lead to cataract in actual eye lens. Our results show that in macromolecular crowding, reminiscent of that of fiber cells in eye lens, moderate intermolecular attraction reduces the osmotic pressure and also prevents the aggregation of proteins of larger sizes induced by depletion force. The crowding factor becomes larger for elongated product shape and for larger relative size of reactant to crowders, which suggests that initial dimerization of crystallins might lead to an avalanche of further associations to larger clusters, however, in order to study this question in more detail, the effects of polydispersity will need to be addressed in future studies. Since many-body interactions are not taken into account, the SPT becomes less convincing at high packing fraction, where crystallization and other types of phase transitions are expected. However, former studies [36, 38, 39] have shown that the osmotic pressure calculated by SPT agrees well with experimental results for various types of globular proteins, when their concentration is below 400 *mg*/*mL*. Our theoretical predictions are thus likely to be relevant for the regime of crystallins in physiological environment, where the concentration is usually 200 ∼ 400 *mg*/*mL* [1, 28].

## Supporting Information

S1 Fig The activity coefficient, *γ*, as a function of packing fraction, *ϕ*. (a) The *γ* − *ϕ* relation for TPM at different average minimum attraction *ϵ*. (b) the *γ* − *ϕ* relation for CBM at different *K* and *n*_*s*_ = 2.
(PDF)

S2 Fig The crowding factor, Γ, as a function of packing fraction, *ϕ*. (a) The Γ − *ϕ* relation for TPM at different average minimum attraction *ϵ*. (b) the *γ* − *ϕ* relation for CBM at different *K* and *n*_*s*_ = 2.
(PDF)

S3 Fig Association of two crystallins in macromolecular crowding for EHM. Sketch of two crystallins as reactants polymerising into a dimer as product for effective hard-sphere model (EHM), where dashed lines and blue spheres respectively represent the actual and effective sizes of crystallins.
(PDF)

S4 FigThe osmotic pressure, Π, as a function of protein concentration, c.The Π − *c* relation for EHM at different $B_{2}^{*}$. Pink hexagons denote experimental results derived from the work of Tardieu *et*. *al*. [15, 37]. In order to compare analytic with experimental results, we set *T* = 298.15*K*.(PDF)

S5 FigThe crowding factor, Γ, as a function of packing fraction, *ϕ*.The ln Γ − *ϕ* relation for EHM at different $B_{2}^{*}$.(PDF)

S6 Fig
$B_{2}^{*}-K$ relation at same activity coefficient.The reduced second virial coefficient, $B_{2}^{*}$, as a function of binding constant, *K*, at different number density of crystallins *ρ*. This relation is obtained under the condition that the activity coefficient, *γ*, derived from EHM equals to that derived from CBM.(PDF)

S7 Fig The crowding factor in weak intermolecular attraction for CBM. The crowding factor, Γ, as a function of packing fraction, *ϕ*, at different *K* for CBM.
(PDF)

S8 Fig Effect of number of binding sites on crowding factor. The crowding factor, Γ, as a function of packing fraction, *ϕ*, at different *α* for CBM with *K* = 0.6.
(PDF)

S9 Fig *ϵ* − K relation at same activity coefficient. The average minimum attraction, *ϵ*, as a function of binding constant, *K*, at different number density of crystallins *ρ*. This relation is obtained under the condition that the activity coefficient, *γ*, derived from TPM equals to that derived from CBM.
(PDF)

S10 Fig Size effect on activity coefficient of crystallins. The activity coefficient, *γ*, as a function of packing fraction, *ϕ*, at different ratio of the diameter of the reactant to that of background crowders, *ς*, for (a) hard spheres, (b) TPM with *ϵ* = 13.9 and (c) CBM with *n*_*s*_ = 2 and *K* = 10.6.
(PDF)

S11 Fig Size effect on crowding factor of crystallins. The crowding factor, Γ, as a function of packing fraction, *ϕ*, at different ratio of the diameter of the reactant to that of background crowders, *ς*, for (a) hard spheres, (b) TPM with *ϵ* = 13.9 and (c) CBM with *n*_*s*_ = 2 and *K* = 10.6.
(PDF)
