# Supplementary material for: The Effect of Attractive Interactions and Macromolecular Crowding on Crystallins Association
Source: PLoS One. 2016 Mar 8;11(3):e0151159. doi: 10.1371/journal.pone.0151159 (PMC4783108; doi:10.1371/journal.pone.0151159)
Supplement: S1 Fig — (a) The γ − ϕ relation for TPM at different average minimum attraction ϵ. (b) the γ − ϕ relation for CBM at different K and ns = 2. (PDF) [file pone.0151159.s001.pdf]

Fig.S1 (a) shows the activity coefficient, γ, for TPM at different average minimum attraction, ϵ. For $\epsilon < 8.0$, the value of $\ln\gamma$ increases with the increase of the packing fraction of crystallins, ϕ. Note that when $\epsilon = 0.0$, we obtain the activity coefficient for hard-spheres based on SPT. For $\epsilon > 13.9$, we find $\ln\gamma$ is negatively correlated with ϕ. However, when $\epsilon \sim 12.0$, we observe that the value of $\ln\gamma$ first decreases slightly and then increases at higher packing fraction at the same ϵ, which reflects the competition of steric repulsion and chemical attraction.

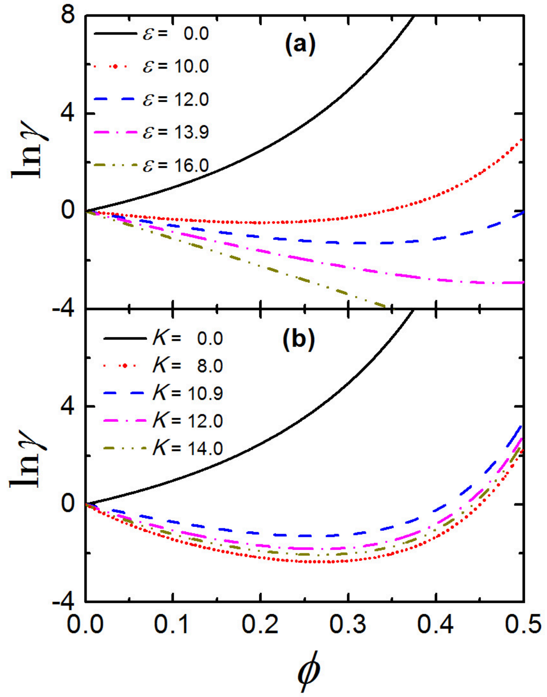

Figure S1. The activity coefficient, γ, as a function of packing fraction, ϕ. (a) The $\gamma - \phi$ relation for TPM at different average minimum attraction ϵ. (b) the $\gamma - \phi$ relation for CBM at different K and $n_s = 2$.

Fig.S1 (b) presents γ at different binding constant, K, for CBM with $n_s = 2$. At same K, with the increase of ϕ, we find $\ln\gamma$ is negative at lower ϕ and positive at higher ϕ, which indicates that the system always favors the adding of another crystallin at lower density after introducing the attractive interactions between particles. At any giving ϕ, the value of $\ln\gamma$ is not sensitive to the change of K, which is quite different from that for TPM. The activity coefficient for hard-spheres is obtained when $K = 0$.
