# Supplementary material for: The Effect of Attractive Interactions and Macromolecular Crowding on Crystallins Association
Source: PLoS One. 2016 Mar 8;11(3):e0151159. doi: 10.1371/journal.pone.0151159 (PMC4783108; doi:10.1371/journal.pone.0151159)
Supplement: S2 Fig — (a) The Γ − ϕ relation for TPM at different average minimum attraction ϵ. (b) the γ − ϕ relation for CBM at different K and ns = 2. (PDF) [file pone.0151159.s002.pdf]

We investigate how crowding factor, Γ, depends on the packing fraction of crystallins, ϕ, for TPM, as illustrated in Fig.S2 (a). For $\epsilon < 8.0$, the value of $\ln\Gamma$ increases with the increase of ϕ at same ϵ, which means that the macromolecular crowding favors the association of the proteins. For $\epsilon > 12.0$, $\ln\Gamma$ is negative and it decreases with the increase of ϕ at same ϵ, which indicates that the macromolecular crowding favors the dissociation of the crystallins. This also denotes that, for $\epsilon = 13.9$ (i.e., system consists of monodisperse crystallins), the crowded environment prevents proteins from polymerization. When $\epsilon = 10.0$, we observe $\ln\Gamma \sim 0.0$ for any given ϕ less than 0.4. This means that for TPM the system is in equilibria with moderate attraction, and such equilibria are unaffected by changing protein concentration.

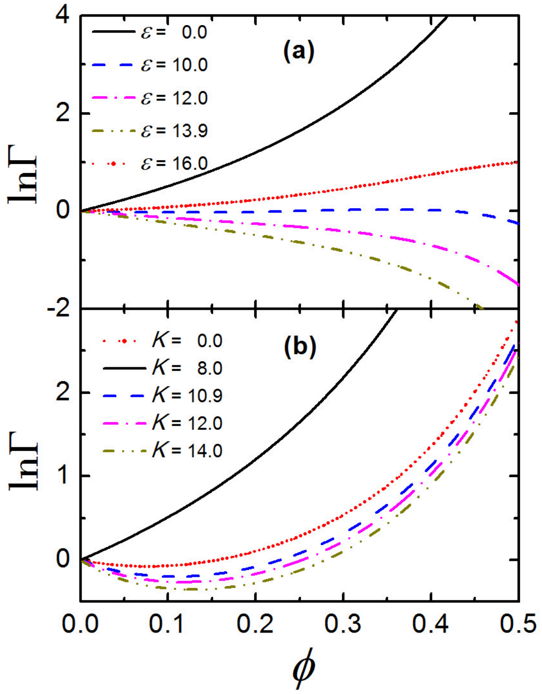

Figure S2. The crowding factor, Γ, as a function of packing fraction, ϕ. (a) the $\Gamma - \phi$ relation for TPM at different average minimum attraction ϵ. (b) the $\Gamma - \phi$ relation for CBM at different K and $n_s = 2$.

The corresponding $\ln\Gamma - \phi$ relation for CBM with $n_s = 2$ is shown in Fig.S2 (b). At fixed ϕ, $\ln\Gamma$ is smaller for larger K, suggesting that the attractive interaction between particles contributes to the disassociation of proteins. Due to the competition between steric repulsion and chemical attraction, for the same K, $\ln\Gamma$ would first decreases at lower ϕ and then increases at higher ϕ, which is different from the prediction of TPM. This means that for CBM the crowding particles work against the association of proteins if the system is highly dense. When $K = 10.6$, for CBM, we obtain $\ln\Gamma \sim 0.0$ in dilute and moderate packing fraction, which confirms that intermolecular attraction can help maintain the association equilibria of crystallins.
