# Supplementary material for: The Effect of Attractive Interactions and Macromolecular Crowding on Crystallins Association
Source: PLoS One. 2016 Mar 8;11(3):e0151159. doi: 10.1371/journal.pone.0151159 (PMC4783108; doi:10.1371/journal.pone.0151159)
Supplement: S3 Fig — Sketch of two crystallins as reactants polymerising into a dimer as product for effective hard-sphere model (EHM), where dashed lines and blue spheres respectively represent the actual and effective sizes of crystallins. (PDF) [file pone.0151159.s003.pdf]

Effective hard-sphere model (EHM) and its analytical results

Theory

Intermolecular interactions have been introduced by Minton *et. al.* [1–4] in the effective hard-sphere model (EHM), where the size of effective particles depends on the magnitude of intermolecular interactions (Fig.S3).

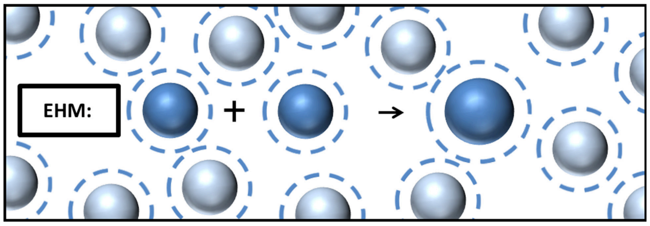

Figure S3. Association of two crystallins in macromolecular crowding for EHM. Sketch of two crystallins as reactants polymerising into a dimer as product for effective hard-sphere model (EHM), where dashed lines and blue spheres respectively represent the actual and effective sizes of crystallins.

Since the SPT only gives γ for hard-spheres, we implement EHM to introduce attractive interaction between crystallins. According to the approximation of EHM [1–3], if the interaction between particles is pairwise additive and isotropic [5], we have

$$\phi' = \frac{cB_2}{8M}, \tag{1}$$

where ϕ' is effective packing fraction, c is protein concentration and $M = 21kDa$ is the molar mass of crystallins. The second virial coefficient, B_2, reflects the pair interaction between crytallins:

$$B_2 = 4\pi N_a \int_0^\infty \left[1 - e^{\frac{-U(r)}{kT}}\right] r^2 dr, \tag{2}$$

where $U(r)$ is the interactive potential, r is the distance between two molecules, N_a is Avogadro number and k is Boltzmann constant.

Therefore, ϕ' changes as a function of interaction between particles. The larger the attraction between proteins, the smaller the value of ϕ' (see Fig.S3). By substituting ϕ' for ϕ in Eq.5 in our main text, we obtain the activity coefficient of crystallins for EHM.

References

1. Minton AP, Edelhoch H. Light scattering of bovine serum albumin solutions: extension of the hard particle model to allow for electrostatic repulsion. Biopolymers. 1982;21(2):451–458.

2. Minton AP. A molecular model for the dependence of the osmotic pressure of bovine serum albumin upon concentration and pH. Biophysical Chemistry. 1995;57(1):65–70.

3. Minton AP. Molecular crowding: Analysis of effects of high concentrations of inert cosolutes on biochemical equilibria and rates in terms of volume exclusion. Methods in Enzymology. 1998;295(1):127–149.

4. Minton AP. Static light scattering from concentrated protein solutions, I: general theory for protein mixtures and application to self-associating proteins. Biophysical Journal. 2007;93(4):1321–1328.

5. Ree FH, Hoover WG. Seventh virial coefficients for hard spheres and hard disks. The Journal of Chemical Physics. 1967;46(11):4181–4197.
