# Supplementary material for: The Effect of Attractive Interactions and Macromolecular Crowding on Crystallins Association
Source: PLoS One. 2016 Mar 8;11(3):e0151159. doi: 10.1371/journal.pone.0151159 (PMC4783108; doi:10.1371/journal.pone.0151159)
Supplement: S4 Fig — The Π − c relation for EHM at different B2*. Pink hexagons denote experimental results derived from the work of Tardieu et. al. [15, 37]. In order to compare analytic with experimental results, we set T = 298.15K. (PDF) [file pone.0151159.s004.pdf]

Effective hard-sphere model (EHM) and its analytical results

Osmotic pressure

The osmotic pressure, Π, of EHM at different B_2^* is given in Fig.S4. It is observed that the value of Π is sensitively dependent on B_2^*, especially at higher protein concentration c. For $B_2^* > -0.2$, the value of Π increases monotonically with the increase of c. For $B_2^* < -0.5$, with the increase of c, the value of Π first increases and then decreases, due to the existence of strong intermolecular attraction. Fig.S4 also shows that experimental results [1,2] of Π are always smaller than Π_i, due to the existence of attractive force between crystallins. We observe that the value of Π at $B_2^* = -0.5$ quantitatively fits well with experimental results.

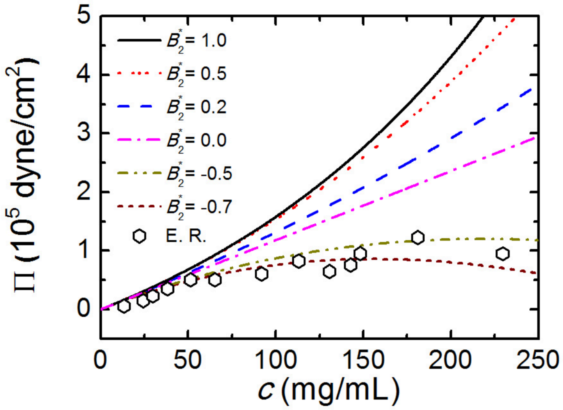

Figure S4. The osmotic pressure, Π, as a function of protein concentration, c. The $\Pi - c$ relation for EHM at different B_2^*. Pink hexagons denote experimental results derived from the work of Tardieu *et. al.* [1,2]. In order to compare analytic with experimental results, we set $T = 298.15K$.

Note that, for CBM, although the existence of intermolecular attraction allows the system to maintain relatively low osmotic pressure, the excluded volume interaction can lead to the increase of Π at very high concentration ($c > 200mg/mL$). Such steric depletion is overlooked for EHM since ϕ' becomes very small or even negative with strong intermolecular attraction.

References

1. Veretout F, Tardieu A. The protein concentration gradient within eye lens might originate from constant osmotic pressure coupled to differential interactive properties of crystallins. European Biophysics Journal. 1989;17(2):61–68.

2. Tardieu A, Veretout F, Krop B, Slingsby C. Protein interactions in the calf eye lens: interactions between β crystallins are repulsive whereas in γ crystallins they are attractive. European Biophysics Journal. 1992;21(1):1–12.
