# Supplementary material for: The Effect of Attractive Interactions and Macromolecular Crowding on Crystallins Association
Source: PLoS One. 2016 Mar 8;11(3):e0151159. doi: 10.1371/journal.pone.0151159 (PMC4783108; doi:10.1371/journal.pone.0151159)
Supplement: S5 Fig — The ln Γ − ϕ relation for EHM at different B2*. (PDF) [file pone.0151159.s005.pdf]

Effective hard-sphere model (EHM) and its analytical results

Crowding factor

Fig.S5 shows the crowding factor, Γ, for EHM at different reduced second virial coefficient, $B_2^* = B_2/B_{hs}$ (where B_{hs} is second virial coefficient for hard-spheres). As shown in Fig.S5, $B_2^* = 0.0$ is the turning point. According to theory of EHM, when $B_2^* = 0.0$, we have $\phi' = 0$ and thus $\ln \gamma \equiv 0$. For $B_2^* > 0.0$, $\ln \Gamma > 0$ and it increases with the increase of ϕ at same B_2^*, which means that the macromolecular crowding favors the association of the proteins. For $B_2^* < 0.0$, the value of $\ln \Gamma$ is negative and it decreases with the increase of ϕ at same B_2^*, which indicates that the macromolecular crowding favors the dissociation of the crystallins. When $B_2^* \sim 0.0$, $\ln \Gamma \sim 0.0$ for any given ϕ. This means that for EHM the system is in equilibria with moderate attraction, and such equilibria are unaffected by changing protein concentration. Note that $\ln \Gamma$ for both TPM and CBM can decrease and then increase at higher protein density with the increase of ϕ which is quite different from the monotonic $\ln \Gamma - \phi$ relation for EHM shown in Fig.S5.

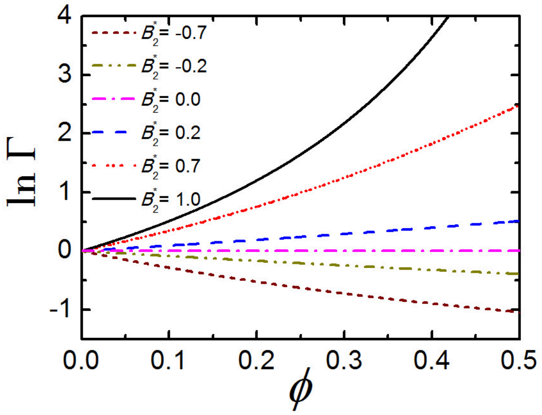

Figure S5. The crowding factor, Γ, as a function of packing fraction, ϕ. The $\ln \Gamma - \phi$ relation for EHM at different B_2^*.
