# Supplementary material for: The Effect of Attractive Interactions and Macromolecular Crowding on Crystallins Association
Source: PLoS One. 2016 Mar 8;11(3):e0151159. doi: 10.1371/journal.pone.0151159 (PMC4783108; doi:10.1371/journal.pone.0151159)
Supplement: S6 Fig — The reduced second virial coefficient, B2*, as a function of binding constant, K, at different number density of crystallins ρ. This relation is obtained under the condition that the activity coefficient, γ, derived from EHM equals to that derived from CBM. (PDF) [file pone.0151159.s006.pdf]

Effective hard-sphere model (EHM) and its analytical results

$B_2^* - K$ relation

The magnitude of the binding constant K and the reduced second virial coefficient B_2^* in EHM are related quantities. In Fig.S6, the relation between both constants, i.e., the values of K and B_2 such that the activity coefficient calculated from both models is the same, is depicted showing an expected linear dependence at low densities and deviations from it in more crowded environment. At larger densities, CBM is more accurate than EHM, since the former reflects the competition between steric repulsion and chemical attraction, while in the EHM the steric effects are not fully taken into account (the effective particle size can even be negative formally). Since many-body effects are not regarded in both models, they are generally applicable in dilute and moderate protein concentrations.

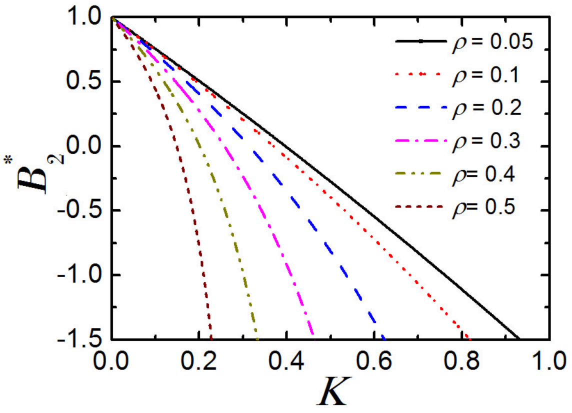

Figure S6. $\mathbf{B_2^* - K}$ relation at same activity coefficient. The reduced second virial coefficient, B_2^*, as a function of binding constant, K, at different number density of crystallins ρ. This relation is obtained under the condition that the activity coefficient, γ, derived from EHM equals to that derived from CBM.
