# Supplementary material for: The Effect of Attractive Interactions and Macromolecular Crowding on Crystallins Association
Source: PLoS One. 2016 Mar 8;11(3):e0151159. doi: 10.1371/journal.pone.0151159 (PMC4783108; doi:10.1371/journal.pone.0151159)
Supplement: S7 Fig — The crowding factor, Γ, as a function of packing fraction, ϕ, at different K for CBM. (PDF) [file pone.0151159.s007.pdf]

The crowding factor in weak intermolecular attraction for CBM

Fig.S7 gives how Γ changes as a function of ϕ for CBM in weak intermolecular attraction with $\alpha = 6$. We observe that, when $K < 0.1$ and $0.3 < \phi < 0.4$, at the same ϕ, $\ln\Gamma$ is larger for larger K. However, in the same region $(0.3 < \phi < 0.4)$, when $K > 0.1$, $\ln\Gamma$ becomes smaller with the increase of K at fixed ϕ. This indicates that at intermediate packing fraction, the dependence of association equilibria of the system on the intermolecular attraction is non-monotonic.

According to theory of CBM, if $\ln\Gamma$ for systems with intermolecular attraction is larger than that for hard-sphere system $(K = 0)$, we have $2\ln\gamma_r^{ch} - \ln\gamma_p^{ch} > 0$, i.e.,

$$-2\alpha S_r \ln\left(1 + K\frac{\gamma_r^{st}\gamma_b^{st}}{\gamma_{r,b}^{st}}\phi\right) > -\alpha S_p \ln\left(1 + K\frac{\gamma_p^{st}\gamma_b^{st}}{\gamma_{p,b}^{st}}\phi\right), \quad (1)$$

where $\gamma_{p,r}^{st}$ is the steric repulsive part of activity coefficient for product-crowder complex. In our case, since $\gamma_r^{st}\gamma_b^{st}/\gamma_{r,b}^{st}$ is always smaller than $\gamma_p^{st}\gamma_b^{st}/\gamma_{p,b}^{st}$ for any giving ϕ, and $S_p \equiv 2^{2/3}S_r$, the above inequality is satisfied only at intermediate packing fraction, where decrease in surface area dominates in its competition with the increase in chemical attraction. This explains why $\ln\Gamma$ for systems with weak intermolecular attraction becomes larger than that for system consisting of hard-spheres.

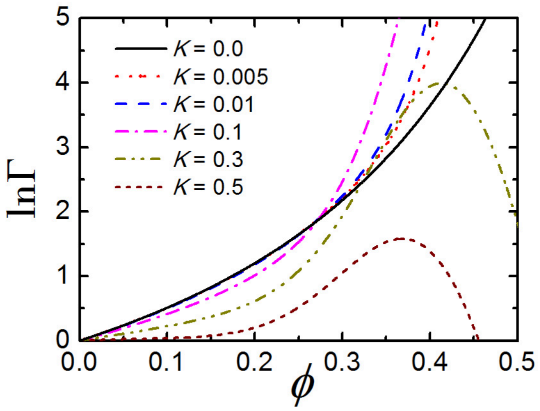

Figure S7. The crowding factor in weak intermolecular attraction for CBM. The crowding factor, Γ, as a function of packing fraction, ϕ, at different K for CBM.
