# Supplementary material for: The Effect of Attractive Interactions and Macromolecular Crowding on Crystallins Association
Source: PLoS One. 2016 Mar 8;11(3):e0151159. doi: 10.1371/journal.pone.0151159 (PMC4783108; doi:10.1371/journal.pone.0151159)
Supplement: S8 Fig — The crowding factor, Γ, as a function of packing fraction, ϕ, at different α for CBM with K = 0.6. (PDF) [file pone.0151159.s008.pdf]

Effect of number of binding sites

Fig.S8 provides how crowding factor Γ changes as a function of packing fraction ϕ at different α for chemical binding model (CBM). We can see that at fixed ϕ, $\ln\Gamma$ decrease with the increase of α. This means that the system favors the dissociation of larger crystallins if we have larger number of binding sites on each protein.

Fig.S8 also shows the competition between steric repulsion and chemical attraction. When α is large, $\ln\Gamma$ would first increases at lower ϕ and then decreases at higher ϕ at fixed K.

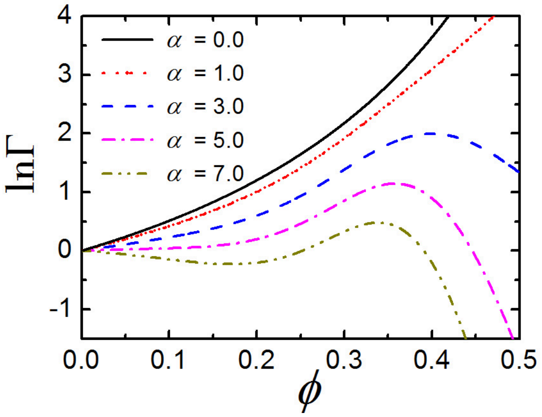

Figure S8. Effect of number of binding sites on crowding factor. The crowding factor, Γ, as a function of packing fraction, ϕ, at different α for CBM with $n_s = 2$ and $K = 10.6$.
