# Supplementary material for: The Effect of Attractive Interactions and Macromolecular Crowding on Crystallins Association
Source: PLoS One. 2016 Mar 8;11(3):e0151159. doi: 10.1371/journal.pone.0151159 (PMC4783108; doi:10.1371/journal.pone.0151159)
Supplement: S9 Fig — The average minimum attraction, ϵ, as a function of binding constant, K, at different number density of crystallins ρ. This relation is obtained under the condition that the activity coefficient, γ, derived from TPM equals to that derived from CBM. (PDF) [file pone.0151159.s009.pdf]

# $\epsilon - $ K relation

We observe the concentration-dependent behavior if we plot $\epsilon$, i.e., the average minimum attraction, as a function of $K$. Fig.S9 gives the $\epsilon - K$ relation at different number density of crystallins $\rho$ under the condition that $\gamma$ derived from TPM equals to that derived from CBM. The $\epsilon - K$ relation is almost linear is in dilute limit, and such linear relation breaks down with the increase of protein density.

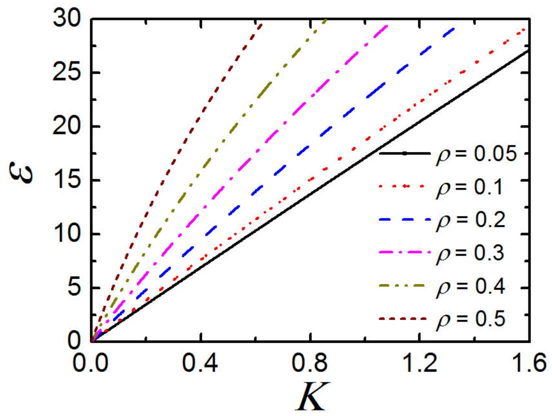


**Figure S9. $\epsilon - $ K relation at same activity coefficient.** The average minimum attraction, $\epsilon$, as a function of binding constant, $K$, at different number density of crystallins $\rho$. This relation is obtained under the condition that the activity coefficient, $\gamma$, derived from TPM equals to that derived from CBM.
