# Supplementary material for: The Effect of Attractive Interactions and Macromolecular Crowding on Crystallins Association
Source: PLoS One. 2016 Mar 8;11(3):e0151159. doi: 10.1371/journal.pone.0151159 (PMC4783108; doi:10.1371/journal.pone.0151159)
Supplement: S10 Fig — The activity coefficient, γ, as a function of packing fraction, ϕ, at different ratio of the diameter of the reactant to that of background crowders, ς, for (a) hard spheres, (b) TPM with ϵ = 13.9 and (c) CBM with ns = 2 and K = 10.6. (PDF) [file pone.0151159.s010.pdf]

## Size effect

Here we analyze how activity coefficient, $\gamma$, changes as a function of the ratio of the diameter of the reactant to that of background crowders, $\varsigma$. Consider background crowders as monodisperse crystallins. The dependence of $\gamma$ on $\varsigma$ for hard spherical system without intermolecular attraction is given in Fig.S10 (a). At the same $\phi$, the value of $\ln\gamma$ is always positive due to steric effect, and it is larger for larger $\varsigma$. Note that to increase the diameter of the reactant protein is tantamount to decrease the size of the crowders at the same $\phi$. Thus, more extra work should be paid on introducing another reactant into system if the reactant is larger, or if the crowding proteins are smaller in size, which is consistent with former study [1].

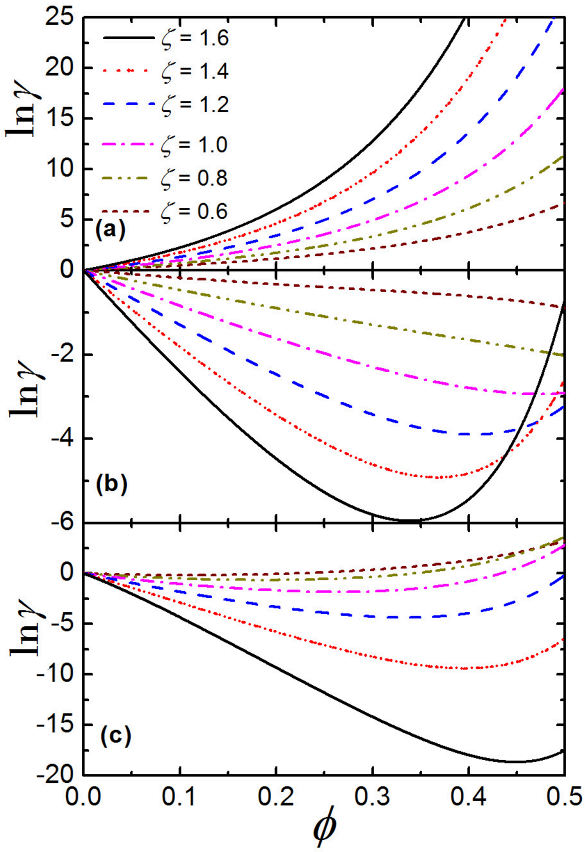


**Figure S10. Size effect on activity coefficient of crystallins.** The activity coefficient, $\gamma$, as a function of packing fraction, $\phi$, at different ratio of the diameter of the reactant to that of background crowders, $\varsigma$, for (a) hard spheres, (b) TPM with $\epsilon = 13.9$ and (c) CBM with $n_s = 2$ and $K = 10.6$.

Quite the contrary, for models that account for the attractive part of interaction, i.e., TPM and CBM, $\ln\gamma$ is inversely correlated with the value of $\varsigma$ at fixed $\phi$ when $\phi < 0.4$, as shown in Fig.S10 (b) and Fig.S10 (c), respectively. Here we simply assume that the diameter of the crowder is fixed to be 1 and change the diameter of reactants (and also product), so that the value of $\varsigma$ in Eq.6 in our paper is changed accordingly. The negative sign indicates that the system favors introducing of another protein. When $\varsigma \geqslant 1.4$, the value of $\ln\gamma$ decreases more rapidly for CBM than that for TPM, with the increase of $\phi$. When $\varsigma < 1.2$, however, the $\ln\gamma - \phi$ relation curve for CBM is

quite similar to that for TPM.

## References

1. Sharp KA. Analysis of the size dependence of macromolecular crowding shows that smaller is better. Proceedings of the National Academy of Sciences. 2015;112(26):7990–7995.
