# Supplementary material for: The Effect of Attractive Interactions and Macromolecular Crowding on Crystallins Association
Source: PLoS One. 2016 Mar 8;11(3):e0151159. doi: 10.1371/journal.pone.0151159 (PMC4783108; doi:10.1371/journal.pone.0151159)
Supplement: S11 Fig — The crowding factor, Γ, as a function of packing fraction, ϕ, at different ratio of the diameter of the reactant to that of background crowders, ς, for (a) hard spheres, (b) TPM with ϵ = 13.9 and (c) CBM with ns = 2 and K = 10.6. (PDF) [file pone.0151159.s011.pdf]

Size effect

Fig.S11 (a) presents the corresponding $\ln\Gamma - \phi$ relation at different ς for system consisting of hard spheres. At fixed ϕ, the value of $\ln\Gamma$ is larger for larger ς. The value of $\ln\Gamma$ is also positively related to ϕ for same ς, which indicates that the surrounding crowders contribute positively to the association of reactants. However, the crowding would work against the association if the attractive part of interaction is taking into account. For instance, based on TPM, as shown in Fig.S11 (b), the value of $\ln\Gamma$ is actually negative and it decreases with the increase of ϕ when $\varsigma > 0.8$, with $\epsilon = 13.9$. Also we observe that, at fixed ϕ, the value of $\ln\Gamma$ is smaller for larger ς for TPM.

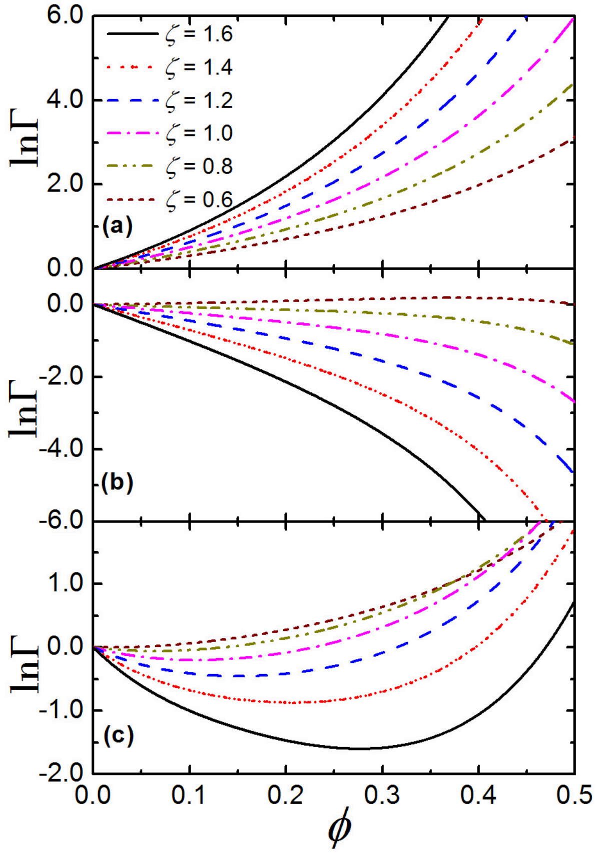

Figure S11. Size effect on crowding factor of crystallins. The crowding factor, Γ, as a function of packing fraction, ϕ, at different ratio of the diameter of the reactant to that of background crowders, ς, for (a) hard spheres, (b) TPM with $\epsilon = 13.9$ and (c) CBM with $n_s = 2$ and $K = 10.6$.

The $\ln\Gamma - \phi$ relation for CBM with $n_s = 2$ and $K = 10.6$ is quite different from that for TPM, see Fig.S11 (c). When $\varsigma < 0.8$, $\ln\Gamma$ is positive and small in magnitude. When $\varsigma > 1.0$, however, $\ln\Gamma$ is negative at small ϕ. This means that the attractive interaction dominates in its competition with steric effect, which makes the environment favor the disassociation of the oligomers of crystallins at low concentration. With the increase of ϕ, the value of $\ln\Gamma$ starts to grow. If ϕ is further increased, we would again obtain positive $\ln\Gamma$. At fixed ϕ, $\ln\Gamma$ is actually larger for smaller ς, which means the presence of the crowders moderately benefits the association of smaller proteins and the dissociation of larger ones. This helps stabilize

the polydispersity of the system.
